# Physical Activity Surveillance Through Smartphone Apps and Wearable Trackers: Examining the UK Potential for Nationally Representative Sampling

**DOI:** 10.2196/11898

**Published:** 2019-01-29

**Authors:** Tessa Strain, Katrien Wijndaele, Søren Brage

**Affiliations:** 1 MRC Epidemiology Unit Institute of Metabolic Science, School of Clinical Medicine University of Cambridge Cambridge United Kingdom

**Keywords:** adult, exercise, fitness trackers, health surveys, smartphone, surveys and questionnaires, United Kingdom, mobile phone

## Abstract

**Background:**

Smartphones and wearable activity trackers present opportunities for large-scale physical activity (PA) surveillance that overcome some limitations of questionnaires or researcher-administered devices. However, it remains unknown whether current users of such technologies are representative of the UK population.

**Objective:**

The objective of this study was to investigate potential sociodemographic biases in individuals using, or with the potential to use, smartphone apps or wearable activity trackers for PA surveillance in the United Kingdom.

**Methods:**

We used data of adults (aged ≥16 years) from two nationally representative surveys. Using the UK-wide 2018 Ofcom Technology Tracker (unweighted N=3688), we derived mutually adjusted odds ratios (ORs; 95% CI) of personal use or household ownership of a smartwatch or fitness tracker and personal use of a smartphone by age, sex, social grade, activity- or work-limiting disability, urban or rural, and home nation. Using the 2016 Health Survey for England (unweighted N=4539), we derived mutually adjusted ORs of the use of wearable trackers or websites or smartphone apps for weight management. The explanatory variables were age, sex, PA, deprivation, and body mass index (BMI). Furthermore, we stratified these analyses by BMI, as these questions were asked in the context of weight management.

**Results:**

Smartphone use was the most prevalent of all technology outcomes, with 79.01% (weighted 2085/2639) of the Technology Tracker sample responding affirmatively. All other outcomes were <30% prevalent. Age ≥65 years was the strongest inverse correlate of all outcomes (eg, OR 0.03, 95% CI 0.02-0.05 for smartphone use compared with those aged 16-44 years). In addition, lower social grade and activity- or work-limiting disability were inversely associated with all Technology Tracker outcomes. Physical inactivity and male sex were inversely associated with both outcomes assessed in the Health Survey for England; higher levels of deprivation were only inversely associated with websites or phone apps used for weight management. The conclusions did not differ meaningfully in the BMI-stratified analyses, except for deprivation that showed stronger inverse associations with website or phone app use in the obese.

**Conclusions:**

The sole use of PA data from wearable trackers or smartphone apps for UK national surveillance is premature, as those using these technologies are more active, younger, and more affluent than those who do not.

## Introduction

National-level physical activity (PA) surveillance usually involves data collection through questionnaires, although some countries also use devices such as accelerometers [1]. The United Kingdom generates prevalence figures from a number of different survey questionnaires; research-grade devices have only been used in small subsamples of some surveys and not yet on a regular basis. Both methods require randomly sampling a proportion of the population to infer representative prevalence and trends; samples are typically small because the resources required are substantial [2]. The representativeness of this sample, however, may be compromised by lower response rates, although a sufficiently large sample size allows advanced statistical modeling to be used to minimize selection bias. It is, therefore, worth considering all surveillance methods that decrease researcher and participant burden, while still achieving large sample sizes. Two such potential options for PA surveillance are smartphone apps and personal wearable activity trackers.

A recent study has demonstrated the potential scale of PA data collection through smartphone apps, describing step count data from 717,527 iPhone users from 111 countries [3]. The combined size and geographical coverage of this dataset make this a potentially useful resource for PA epidemiology. However, the sample was restricted to iPhone users, who may not be representative of the general population. Unsurprisingly, most of the data originated from people living in richer countries. Among the 46 countries for which demographic data were presented, the median age was under 40 years, and there was a strong tendency toward overrepresentation of men. Such demographic selection biases would be problematic for global and national surveillance unless they were taken into account in the analyses.

The aim of this study was to investigate potential sociodemographic biases in individuals using, or with the potential to use, smartphone apps or wearable activity trackers for PA surveillance in the United Kingdom.

## Methods

### Data Sources

We used two nationally representative surveys that collected data relating to the use of smartphone apps or wearable activity trackers: the 2018 Ofcom Technology Tracker (TT) survey and the 2016 Health Survey for England (HSE); the former covered all 4 home nations in the United Kingdom, while the latter covered England only.

The 2018 TT data were obtained on May 18, 2018, through contact with Ofcom but have since been made publicly available on their website [4]. The 2016 HSE data were downloaded from the UK Data Archive on April 17, 2018 [5].

### Ofcom Technology Tracker

The Ofcom TT survey measures awareness, access, use of, and attitudes toward fixed and mobile telecoms, internet, multichannel television, and radio of adults (aged ≥16 years) in the United Kingdom [6]. The 2018 survey was run by Saville-Rossiter Base on behalf of Ofcom, the UK communications regulator [7]. Data were collected between January 3 and February 28, 2018, by interviewer-led, tablet computer-assisted interviews carried out at respondents’ homes. A quota sample of 3730 adults was selected to match the 2011 Census data on age, sex, and social grade [8]. Weighting matched the sample to the geographical and demographic population profile of the United Kingdom [6].

#### Device Ownership and Use

Two main outcomes were derived from the responses to questions on device use:

1. *Personal use of a smartphone*. Respondents were provided with the following description: “a smartphone is a phone on which you can easily access emails, download files and applications, as well as view websites and generally surf the internet. Popular brands of smartphone include BlackBerry, iPhone, and Android phones such as the Samsung Galaxy S6.”
2. *Personal use of “a smartwatch or wearable tech such as fitness trackers*.” The following description was provided: “a wearable computer that may be compatible with a smartphone. Brands include Apple Watch, Pebble, Fitbit, and Garmin.”

In addition, we derived “household ownership of a smartwatch or fitness tracker” as a supplementary outcome to identify any differences between ownership and use.

#### Explanatory Variables

Respondents reported their age in years and a 3-category variable was derived: 16-44, 45-64, and ≥65 years. Sex was coded by the interviewer but not asked directly of the respondent. Social grade was prederived on the dataset according to the National Readership Survey categories [8]. This was based on the self-reported occupational details of the main earner in the household: position or rank, industry, qualifications, and the number of staff members responsible for. The commonly used 2-category variable was derived—ABC1: higher; intermediate, supervisory, or junior managerial, administrative, or professional occupations and C2DE: skilled manual, semiskilled or unskilled manual workers, state pensioners, casual or lowest-grade workers, or unemployed with state benefits only. Respondents who self-reported any of the following conditions were deemed to have an activity or work-limiting disability: breathlessness or chest pains, visual, hearing, mobility, speaking or communicating difficulties, limited ability to reach, mental health problems, dyslexia, or any other self-reported health problems that limit daily activities or work. Postcodes were not included on the dataset, but 2 geographical variables were prederived from them: urban or rural location and UK home nation. Rural was defined as a postcode in villages with a population <2000 that are at least 10 miles from a town or city with a population >15,000. All other locations were defined as urban.

#### Statistical Analysis

The analysis sample consisted of 3688 individuals who provided complete data for all relevant variables. Logistic regressions were used to calculate the crude and mutually adjusted (for age, sex, social grade, disability status, urban or rural, and UK home nation) odds ratios (ORs) for the likelihood of reporting (1) personal use of a smartphone; (2) personal use of a wearable tracker; and (3) household ownership of a wearable tracker. All analyses were weighted using the sampling weights provided.

### The 2016 Health Survey for England

The HSE is an annual survey commissioned by the Health and Social Care Information Centre, undertaken by the NatCen Social Research and University College London [9]. It aims to provide nationally representative data on the prevalence and trends of health conditions and behaviors for the population living in private households in England.

The majority of information, including the demographic data, was collected through a computer-assisted, interviewer-led interview carried out at respondents’ homes, spread throughout the year [10]. In addition, respondents’ height and weight were measured at the main interview. A follow-up visit by a nurse was offered to all participants. This consisted of a further questionnaire, including items on the use of technology for weight management, more anthropometric measurements, and a blood sample. The questions about technology use were relevant to the present analysis (see below). A total of 5049 adults (aged ≥16 years) participated in both the main interview and nurse visit. Sampling weights were provided for this subsample that accounted for selection probability and nonresponse bias, calibrating to mid-year population estimates for sex and age groups by region. Further details are available elsewhere [9].

#### Use of Technology for Weight Management

As part of the nurse visit, respondents were asked whether they had used any devices or services to help manage or change their weight (multiple responses allowed). The 2 responses of interest were (1) activity trackers or fitness monitors such as a Fitbit, FuelBand, or Jawbone Up and (2) websites or mobile phone apps. For the activity tracker question, nurses were given the prompt “explain if necessary, activity trackers or fitness monitors are often a band worn on the wrist like a watch. They keep track of the number of steps people take and track activity over time” [10].

#### Explanatory Variables

Age, sex, and PA in the 28 days prior to the interview were reported. The following 3-category variable for age was derived: 16-44, 45-64, and ≥65 years. We used the prederived variable on compliance to the UK Chief Medical Officers’ PA recommendation of inactive (0-<150 minutes/week) and active (≥150 minutes/week) [11]. This was derived from questions on the duration and frequency of different domains of PA according to the protocol used to derive the national prevalence estimates. All heavy housework, heavy manual nonoccupational activity, gardening, and do-it-yourself home maintenance were counted as a moderate-intensity activity; examples of activities were provided to assist participants identify whether an activity was intense enough. Time spent climbing stairs or ladders, lifting, carrying or moving heavy loads, and walking at work was reported but only counted as moderate intensity if the respondents’ Standard Occupational Classification 2000 code was in a predetermined list [12,13]. Sport and exercise activities were counted as a moderate or vigorous activity dependent on a predetermined list, which, for some activities, factored in response to a question as to whether it made them out of breath or sweaty. For those aged <65 years, walking counted as moderate intensity if the self-reported pace was “fairly brisk” or “fast pace—at least 4 miles per hour.” All walking counted as moderate-intensity activity in those aged ≥65 years. The total weekly duration of vigorous intensity activity was counted as double that of moderate intensity and summed to give a total that was used to determine compliance with the PA recommendation.

The body mass index (BMI; weight, kg/height, m^2^) was calculated using the measurements obtained at the main interview. A 3-category variable was derived: normal or underweight (<25 kg/m^2^), overweight (25-<30 kg/m^2^), and obese (≥30 kg/m^2^). A score on the 2015 Index of Multiple Deprivation (a multidomain measure of area deprivation [14]) was prederived from respondents’ postcodes. Quintiles of this score (based on the main interview sample) were provided on the downloaded dataset. We derived a binary variable to identify the most deprived 20%.

#### Statistical Analysis

The analysis sample consisted of 4539 individuals who provided complete data for all relevant variables. Logistic regressions were used to calculate the crude and mutually adjusted (for age, sex, activity status, deprivation status, and BMI) ORs for the likelihood of reporting the use of (1) an activity tracker or fitness monitor and (2) a website or mobile phone app, for weight management. All analyses were weighted using the sampling weights provided. As these questions were asked in the context of weight management, and our interest here is more generic activity tracking, we also ran the analyses stratified by BMI category.

## Results

### Sample Characteristics and Prevalence of Activity Tracking Technology

**Figure 1:**
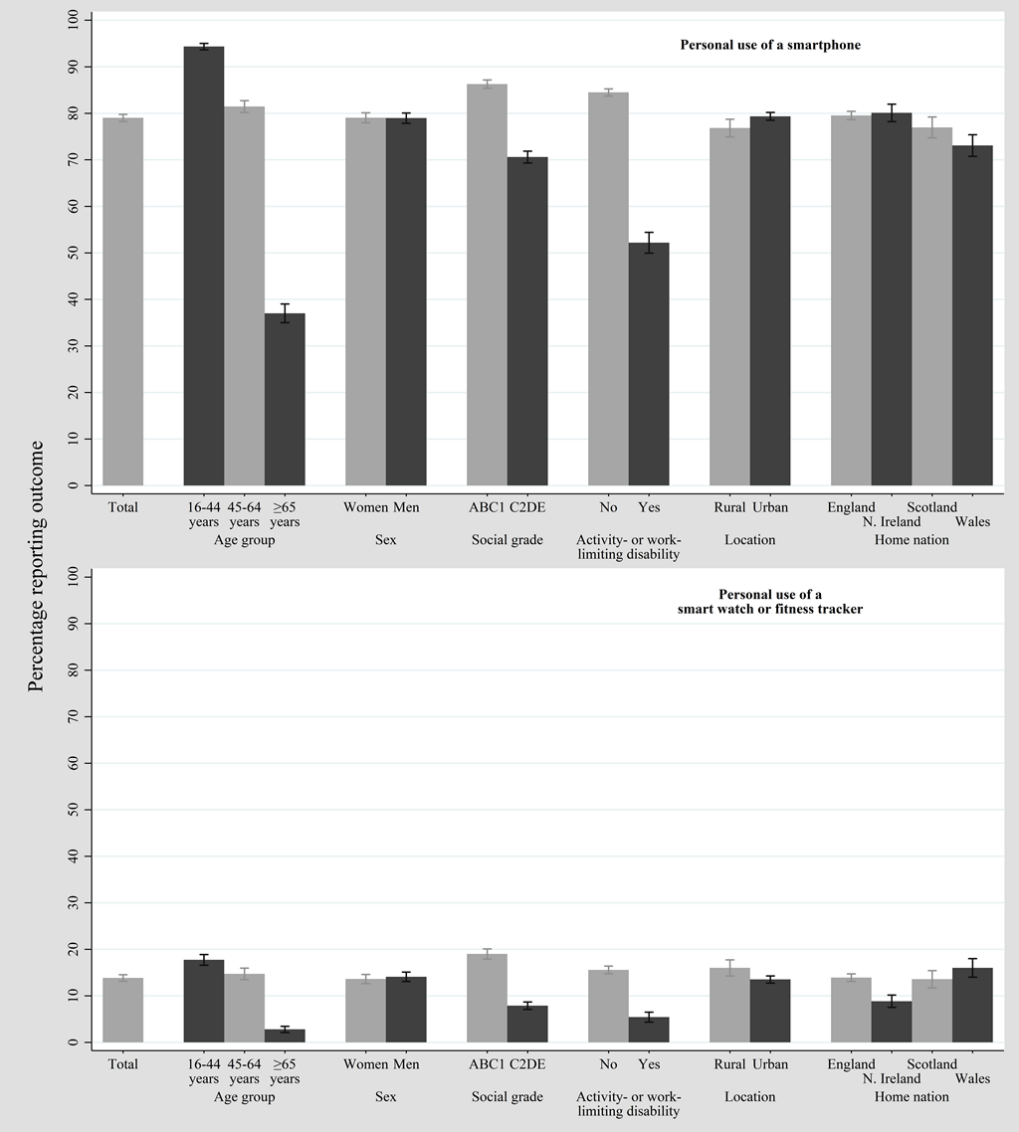
Percentage reporting the use of activity tracking-related technology in the 2018 Ofcom Technology Tracker survey (unweighted N=3688, weighted N=2639).

**Figure 2:**
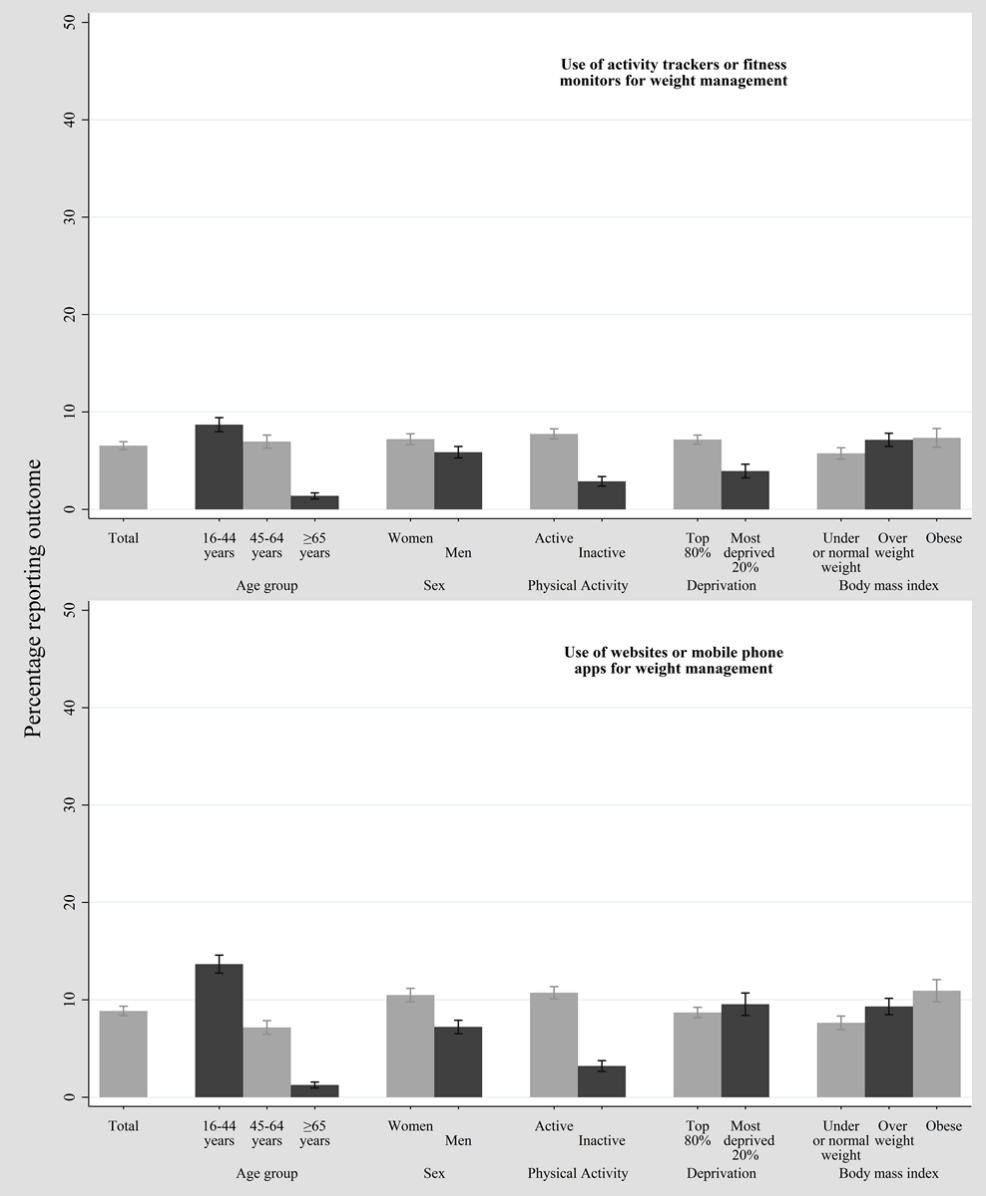
Percentage reporting the use of activity tracking-related technology in the 2016 Health Survey for England (unweighted N=4539, weighted N=4380).

Tables 1 and 2 show the sociodemographic characteristics of weighted TT and HSE samples, respectively (see Multimedia Appendix 1 for the BMI-stratified HSE sample data). Figure 1 and Multimedia Appendix 2 show that smartphone use was the most prevalent of all the investigated TT outcomes (weighted 2085/2639, 79.01%). Prevalence of personal use of a smartwatch or fitness tracker was 13.86% (weighted 366/2639). Those aged ≥65 years, those who had an activity- or work-limiting disability, or those with a lower social grade reported the lowest prevalence figures. Prevalence of household ownership of a smartwatch or fitness tracker was slightly higher than that for personal use but followed a similar pattern among subgroups (see Multimedia Appendix 3). Data from the HSE (Figure 2 and Multimedia Appendix 4) showed that 6.53% (weighted 286/4380) of the sample reported using a wearable tracker for weight management and 8.86% (weighted 388/4380) of the sample reported using websites or phone apps for weight management.

### Sociodemographic Correlates of Activity Tracking Technology Use in the 2018 Technology Tracker

**Figure 3:**
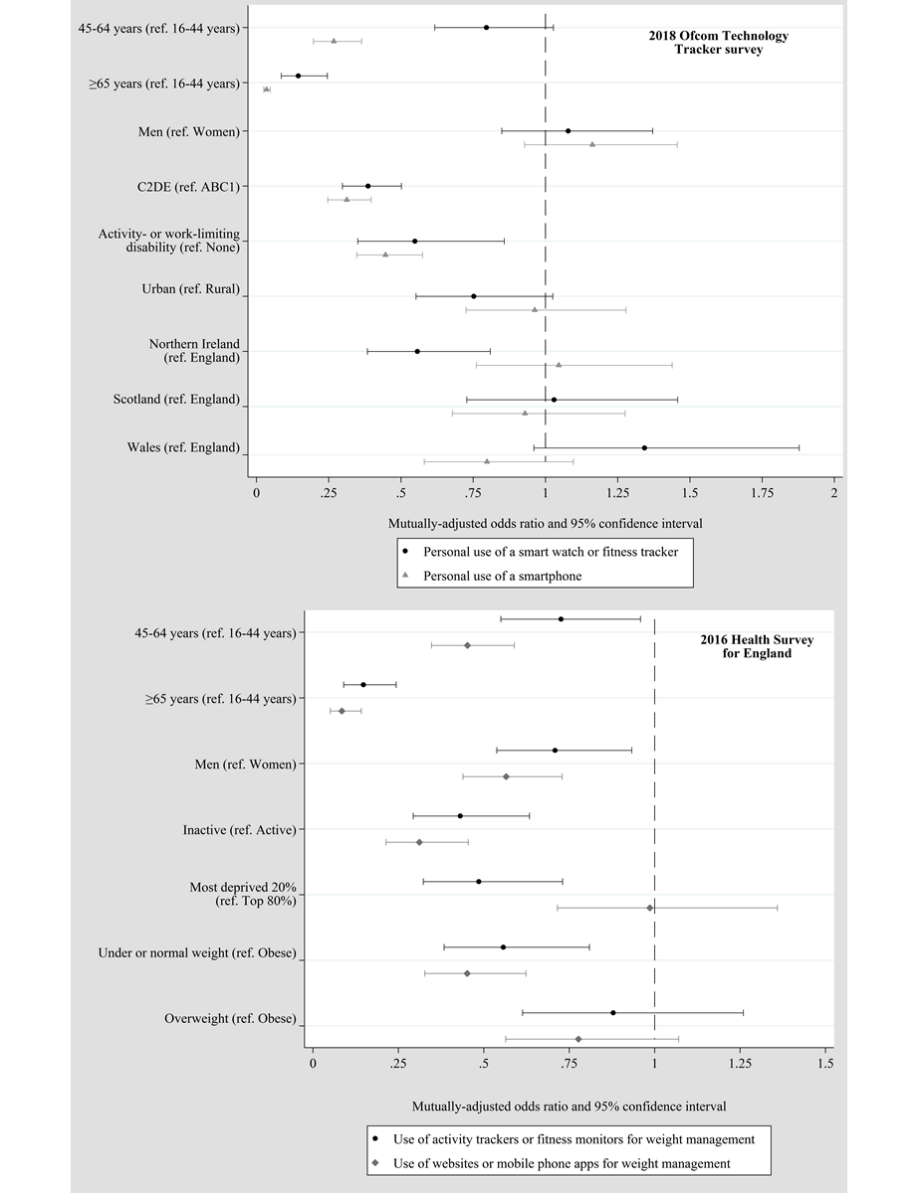
Mutually adjusted odds ratios of reporting the use or ownership of activity tracking-related technology by sociodemographic characteristics in the 2018 Ofcom Technology Tracker survey (unweighted N=3688, weighted N=2639) and the 2016 Health Survey for England (unweighted N=4539, weighted N=4380).

Figure 3 shows that age ≥65 years is the characteristic associated with the lowest odds of personal use of a smartwatch or fitness tracker, as well as of the personal use of a smartphone in the TT survey. The mutually adjusted ORs for this group compared with those aged 16-44 years were 0.14 (95% CI 0.09-0.24) and 0.03 (95% CI 0.02-0.05), respectively. In addition, age between 45 and 64 years was associated with a lower likelihood of reporting smartphone use (mutually adjusted OR 0.27, 95% CI 0.20-0.36) but the respective OR CI for personal use of a smartwatch or fitness tracker just crossed one.

Lower social grade (C2DE compared with ABC1) was inversely associated with the use of tracking technology, with mutually adjusted ORs ranging between 0.31 and 0.42 (see Multimedia Appendix 2). Reporting an activity- or work-limiting disability inversely correlated with the personal use of a smartwatch or fitness tracker and smartphone use (mutually adjusted ORs 0.55, 95% CI 0.35-0.86 and 0.45, 95% CI 0.35-0.57, respectively). There were mixed results regarding the geographical explanatory variables of urban-rural and home nation; those in urban areas were less likely to own a smartwatch or fitness tracker in the household (mutually adjusted OR 0.69, 95% CI 0.53-0.90), but the CIs crossed one for the other outcomes. Those living in Northern Ireland were less likely to report personal use of a smartwatch or fitness tracker than those living in England (mutually adjusted OR 0.56, 95% CI 0.38-0.81). Other comparisons between nations and for other outcomes did not present clear patterns. We observed no differences by sex for any outcome. Furthermore, there were no substantial differences between the results for personal use and household ownership of a smart watch or activity tracker (see Multimedia Appendix 5).

**Table 1:** Sociodemographic profile of the 2018 Ofcom Technology Tracker sample (unweighted N=3688, weighted N=2639).

Characteristics		Sample, weighted n (%)	SE
**Age group (years)**			
	16-44	1259 (47.72)	1.0
	45-64	869 (32.93)	0.9
	>65	511 (19.35)	0.7
**Sex**			
	Women	1350 (51.17)	1.0
	Men	1289 (48.83)	1.0
**Social grade^a^**			
	ABC1	1417 (53.69)	1.0
	C2DE	1222 (46.31)	1.0
**Disability status**			
	No activity or work-limiting disability	2192 (83.05)	0.7
	Activity or work-limiting disability	447 (16.95)	0.7
**Location**			
	Rural	351 (13.29)	0.6
	Urban	2288 (86.71)	0.6
**UK home nation**			
	England	2201 (83.42)	0.6
	Northern Ireland	73 (2.78)	0.1
	Scotland	232 (8.81)	0.5
	Wales	132 (4.99)	0.3

^a^ABC1 includes those where the main household earner is in a higher, intermediate, supervisory, or junior managerial, administrative, or professional occupation and C2DE includes those where the main household earner is a skilled manual, semiskilled or unskilled manual worker, state pensioner, casual or lowest-grade worker, or unemployed with state benefits only.

**Table 2:** Sociodemographic profile of the 2016 Health Survey for England sample (unweighted N=4539, weighted N=4380).

Characteristic		Sample, weighted n (%)	SE
**Age group (years)**			
	16-44	2015 (45.99)	0.9
	45-64	1408 (32.14)	0.7
	>65	958 (21.86)	0.6
**Sex**			
	Women	2198 (50.19)	0.8
	Men	2182 (49.81)	0.8
**Physical activity**			
	Active	3292 (75.15)	0.7
	Inactive	1088 (24.85)	0.7
**Deprivation**			
	Top 80%	3539 (80.79)	0.7
	Most deprived 20%	841 (19.21)	0.7
**Body mass index**			
	Under or normal weight	2013 (46.96)	0.8
	Overweight	1506 (34.39)	0.8
	Obese	861 (19.65)	0.6

### Sociodemographic Correlates of Activity Tracking Technology Use in the 2016 Health Survey for England

In the 2016 HSE, age ≥65 years showed the strongest inverse relationships with the use of tracking technology, with mutually adjusted ORs between 0.08 and 0.15 (Figure 3; Multimedia Appendix 4). In addition, not meeting the PA guidelines (compared with meeting them) and male sex (compared with female) were inversely associated with both uses of technology for weight management (mutually adjusted ORs 0.31-0.43 and 0.57-0.71, respectively). Those in the 20% most deprived areas were less likely to report using websites or phone apps for weight management compared with those in the top 80%; however, there was no evidence of a difference in the use of wearable trackers for weight management. Conversely, those aged 45-64 years were less likely to use a wearable tracker for weight management compared with those aged 16-44 years; however, there were no differences in the website or phone app use. A majority of the conclusions did not differ meaningfully when the analyses were stratified by BMI. One notable exception was area deprivation, which showed stronger inverse associations with the smartphone use in the obese individuals (see Multimedia Appendices 6 and 7).

## Discussion

### Principal Findings

This is the first study to consider the issue of representativeness of users of tracking technology in UK data in relation to PA surveillance. This is timely as the Expert Group reviewing the UK PA guidelines has recommended that all technological advances in the field of PA measurement are considered for the long-term future of surveillance (report due to be published in 2019).

Our results show that users or owners of smartphones and wearable activity trackers are not representative of the general population in the United Kingdom; this was also true for the use of wearable activity trackers or websites or apps in the context of weight management. Statistical weighting, that is, attempting to make the sample results more reflective of the population distribution of key sociodemographic variables, is unlikely to be able to resolve these issues for 2 reasons. First, our results indicate that PA levels themselves may be correlated with the use of such technologies, albeit it is important to note that these data from the HSE are asked within the context of weight management. If users are more active than nonusers, adjusting for other population demographic characteristics will still lead to an overestimate. Second, some of the biases are strong (eg, age >65 years), meaning that certain sample substrata would be weighted heavily and be highly influential in the estimates. When such a minority of a population use the technology required for measurement, such as would be the case for some subgroups, it is unlikely that the assumption that users and nonusers are similar with respect to the relevant characteristics would hold. Further discussion on the issues of statistical weighting in population surveys is provided elsewhere [15].

### Comparison With Prior Work

Despite smartphone usage being almost ubiquitous among people aged 16-44 years, it remains much less common in those aged >65 years, at around one-third. Age ≥65 years was the strongest inverse correlate for all outcomes. This is comparable to similar studies looking at smartphone use undertaken in Canadian [16], Swiss [17], German [18], and American [19] samples. These studies also found differences by activity levels [17-19], some indications of health status [16], and measures of socioeconomic position [16,18,19].

This is a fast-moving field, and trends indicate that activity trackers will become more prevalent in the coming years. The Ofcom TT data indicate that the percentage reporting using a smartwatch has increased from 2% in 2015 to 14% in 2018 [20,21]. The percentage of people aged ≥55 years (no older age group breakdown available) using smartphones has increased from 32% to 51% over that period [20,21]. Although it is hard to reach conclusions with such small starting prevalence figures, it does appear that it is the more affluent driving the increase, but that it is relatively uniform across the age groups [20,21]. As more data are collected, this will be an important trend to monitor.

### Strengths and Limitations

A major strength of this analysis is that it uses the most up-to-date nationally representative data. Both datasets were only released in April 2018; the Ofcom TT data were even collected this year. Although the HSE questions were asked in a weight management context, they are the only source of data to provide paired information on PA and device use. We examined this potential bias by performing BMI-stratified sensitivity analyses (see Multimedia Appendices 6 and 7). The results were similar between weight groups, except for deprivation where the inverse association was stronger in the obese.

The PA levels in the HSE “nurse interview sample” were higher than reported for the “main interview” sample in 2016 (58% women and 66% of men [13]), even after weighting by age, sex, and geographical location. This bias is likely to affect (overestimate) the prevalence estimates for those using wearable trackers or websites or mobile phone apps for weight management. For our specific purpose, it would have been advantageous for the TT survey to also have included a measure of PA, as that is the potential bias that most limits the use of this technology for PA surveillance.

A limitation of this study was that we were only able to investigate differences in the use or ownership due to the questions asked in the surveys. Ownership of a smartphone will not necessarily mean that an individual is willing to download and use an activity tracking app and then share the data for national surveillance purposes. Even among willing individuals, there may be further biases concerning what activities are recorded: for example, a smartphone app is unlikely to be used to record swimming, and wrist-worn devices may not be able to adequately quantify activity when cycling. This may also be influenced by how they are worn (eg, trouser or breast pocket, handbag). As both the types of activities that adults participate in and the method of wearing a smartphone have been shown to differ systematically by characteristics such as sex, age, disabilities, and cultural norms [22,23], this is another layer of representativeness that should be considered. In addition, we were unable to examine why people were using devices in the TT survey, whereas in the HSE survey, the questions were only asked for weight management. Reportedly, many who use these devices do so to improve their health [24]. This may mean that individuals’ behavior while using a device is not representative of their habitual levels. More detailed data will be needed to understand whether this introduces further or different biases. Ideally, we should also have been able to identify users of different smartphone operating systems (asked in the TT survey but not on the publicly available dataset), as this can have a bearing on what apps are available for download and the practicalities of obtaining the data for researchers. Furthermore, data on the use of specific PA tracking apps would have added useful information. This investigation does not allow us to make any conclusions regarding the validity of these technologies for measuring the metrics of PA. This issue is equally important when considering their potential use for PA surveillance, particularly as some evidence suggests that there may be systematic biases for some estimates. For example, walking metrics, such as step count and distance, appear to be underestimated at slower speeds, higher BMI, female sex, and among certain ethnic groups [25,26]. Finally, the scope of this study was to consider these data sources for surveillance purposes. Other study designs, most notably those using smartphones and activity trackers as an intervention aids for changing PA, may well conclude that these methods have utility [27]. In addition, the study of within-person patterns across the week or year using these data sources may well generalize better to the general population, but no data are currently allowing us to examine that.

### Conclusions

We conclude that the sole use of PA data from personal trackers or smartphone apps for national surveillance in the United Kingdom is premature as those using these devices are more active, younger, and more affluent than those who do not.

## Appendix

Multimedia Appendix 1 Sociodemographic profile of the 2016 Health Survey for England sample (unweighted N=4539, weighted N=4380), stratified by body mass index.

Multimedia Appendix 2 Crude and mutually-adjusted odds ratios of reporting personal use of a smart watch or fitness tracker, household ownership of a smart watch or fitness tracker, or personal use of a smartphone, by socio-demographic characteristic in the 2018 Ofcom Technology Tracker survey (unweighted N=3688, weighted N=2639).

Multimedia Appendix 3 Percentage reporting household ownership of a smart watch or activity tracker in the 2018 Ofcom Technology Tracker survey (unweighted N=3688, weighted N=2639).

Multimedia Appendix 4 Crude and mutually-adjusted odds ratios of reporting use of activity trackers or fitness monitors or websites or mobile phone applications for weight management, by socio-demographic characteristic in the 2016 Health Survey for England (unweighted N=4539, weighted N=4380).

Multimedia Appendix 5 Mutually-adjusted odds ratios of reporting household ownership of smart watch or activity tracker by socio-demographic characteristic, in the 2018 Ofcom Technology Tracker survey (unweighted N=3688, weighted N=2639).

Multimedia Appendix 6 Crude and mutually-adjusted odds ratios of reporting use of activity trackers or fitness monitors or websites or mobile phone applications for weight management, by sociodemographic characteristic, stratified by body mass index, in the 2016 Health Survey for England (unweighted N=4539, weighted N=4380).

Multimedia Appendix 7 Mutually-adjusted odds ratios of reporting use of activity trackers or fitness monitors or websites or mobile phone applications for weight management, by sociodemographic characteristic, stratified by body mass index, in the 2016 Health Survey for England (unweighted N=4539, weighted N=4380).

## Abbreviations

BMI: body mass index
HSE: Health Survey for England
OR: odds ratio
PA: physical activity
TT: Technology Tracker
