# Supplementary material for: Physical Activity Surveillance Through Smartphone Apps and Wearable Trackers: Examining the UK Potential for Nationally Representative Sampling
Source: JMIR Mhealth Uhealth. 2019 Jan 29;7(1):e11898. doi: 10.2196/11898 (PMC6371078; doi:10.2196/11898)
Supplement: Multimedia Appendix 1 [file mhealth_v7i1e11898_app1.pdf]

Multimedia Appendix 1. Socio-demographic profile of the 2016 Health Survey for England sample (unweighted N=4539, weighted N=4380), stratified by body mass index.

		Under/normal weight	Overweight	Obese
		Weighted n (%)	Weighted n (%)	Weighted n (%)
		SE	SE	SE
**Age group**				
	16-44 years	1117 (55.48)	583 (38.69)	315 (36.58)
		1.2	1.5	1.8
	45-64 years	513 (25.46)	555 (36.86)	340 (39.51)
		1.0	1.3	1.7
	65+ years	384 (19.05)	368 (24.45)	206 (23.91)
		0.8	1.0	1.4
**Sex**				
	Women	1114 (55.35)	622 (41.28)	462 (53.68)
		1.3	1.4	1.8
	Men	899 (44.65)	884 (58.72)	399 (46.32)
		1.3	1.4	1.8
**Physical activity**				
	Active	1613 (80.11)	1131 (75.09)	548 (63.65)
		1.0	1.2	1.7
	Inactive	400 (19.89)	375 (24.91)	313 (36.35)
		1.0	1.2	1.7
**Deprivation**				
	Top 80%	1695 (84.20)	1210 (80.34)	634 (73.61)
		0.9	1.2	1.5
	Most deprived 20%	318 (15.80)	296 (19.66)	227 (26.39)
		0.9	1.2	1.5
