# Supplementary material for: Physical Activity Surveillance Through Smartphone Apps and Wearable Trackers: Examining the UK Potential for Nationally Representative Sampling
Source: JMIR Mhealth Uhealth. 2019 Jan 29;7(1):e11898. doi: 10.2196/11898 (PMC6371078; doi:10.2196/11898)
Supplement: Multimedia Appendix 2 [file mhealth_v7i1e11898_app2.pdf]

Multimedia Appendix 2. Crude and mutually-adjusted odds ratios of reporting personal use of a smart watch or fitness tracker, household ownership of a smart watch or fitness tracker, or personal use of a smartphone, by socio-demographic characteristic in the 2018 Ofcom Technology Tracker survey (unweighted N=3688, weighted N=2639).

	Weighted n (%) reporting outcome	Crude odds ratio	Mutually-adjusted odds ratio
	SE	95% confidence interval	95% confidence interval
		P-value	*P*-value
**Outcome: Personal use of a smart watch or fitness tracker**			
**Age group**			
16-44 years	223 (17.74)	ref	ref
	1.1		
45-64 years	128 (14.72)	0.80	0.80
	1.2	(0.62,1.03)	(0.62,1.03)
		P = .08	*P* = .08
65+ years	14 (2.79)	*0.13*	*0.14*
	0.7	*(0.08,0.22)*	*(0.09,0.24)*
		P < .001	*P < .001*
**Sex**			
Women	184 (13.62)	ref	ref
	1.0		
Men	182 (14.10)	1.04	1.08
	1.0	(0.83,1.31)	(0.85,1.37)
		P = .73	*P* = .54
**Social grade**			
ABC1	269 (19.00)	ref	ref
	1.1		
C2DE	96 (7.89)	*0.37*	*0.39*
	0.8	*(0.28,0.47)*	*(0.30,0.50)*
		P < .001	*P < .001*
**Disability status**			
No activity or work limiting disability	341 (15.57)	ref	ref
	0.8		
Activity or work limiting disability	24 (5.43)	*0.31*	*0.55*
	1.1	*(0.20,0.48)*	*(0.35,0.86)*
		P < .001	*P = .008*
**Location**			
Rural	56 (16.01)	ref	ref
	1.7		

	Urban	309 (13.52)	0.82	0.75
		0.8	(0.62,1.09)	(0.55,1.03)
			P = .17	*P* = .07
**UK home nation**				
	England	306 (13.92)	ref	ref
		0.8		
	Northern Ireland	6 (8.84)	*0.60*	*0.56*
		1.3	*(0.42,0.85)*	*(0.38,0.81)*
			P = .004	*P = .002*
	Scotland	31 (13.57)	0.97	1.03
		1.9	(0.69,1.36)	(0.73,1.46)
			P = .86	*P* = .87
	Wales	21 (16.02)	1.18	1.34
		2.0	(0.86,1.62)	(0.96,1.88)
			P = .31	*P* = .09
**Outcome: Household ownership of a smart watch or fitness tracker**				
**Age group**				
	16-44 years	321 (25.52)	ref	ref
		1.3		
	45-64 years	193 (22.16)	0.83	0.81
		1.4	(0.67,1.03)	(0.65,1.01)
			P = .09	*P* = .06
	65+ years	28 (5.52)	*0.17*	*0.17*
		1.0	*(0.12,0.25)*	*(0.12,0.26)*
			P < .001	*P < .001*
**Sex**				
	Women	272 (20.13)	ref	ref
		1.1		
	Men	270 (20.98)	1.05	1.10
		1.2	(0.87,1.28)	(0.89,1.34)
			P = .60	*P* = .38
**Social grade**				
	ABC1	382 (26.93)	ref	ref
		1.2		
	C2DE	161 (13.14)	*0.41*	*0.42*
		1.0	*(0.33,0.51)*	*(0.34,0.53)*
			P < .001	*P < .001*
**Disability status**				
	No activity or work limiting disability	493 (22.51)	ref	ref
		0.9		

	Activity or work limiting disability	49 (10.93)	*0.42*	0.71
		1.5	*(0.31,0.58)*	(0.50,1.01)
			P < .001	*P* = .06
**Location**				
	Rural	87 (24.84)	ref	ref
		2.1		
	Urban	455 (19.88)	*0.75*	*0.69*
		0.9	*(0.59,0.96)*	*(0.53,0.90)*
			P = .02	*P = .006*
**UK home nation**				
	England	449 (20.38)	ref	ref
		0.9		
	Northern Ireland	11 (14.69)	*0.67*	*0.61*
		1.7	*(0.51,0.89)*	*(0.45,0.83)*
			P = .006	*P = .002*
	Scotland	54 (23.36)	1.19	1.26
		2.3	(0.90,1.57)	(0.94,1.69)
			P = .22	*P* = .13
	Wales	28 (21.50)	1.07	1.19
		2.2	(0.81,1.42)	(0.89,1.59)
			P = .64	*P* = .25
**Outcome: Personal use of a smartphone**				
**Age group**				
	16-44 years	1188 (94.35)	ref	ref
		0.7		
	45-64 years	708 (81.44)	*0.26*	*0.27*
		1.3	*(0.19,0.35)*	*(0.20,0.36)*
			P < .001	*P < .001*
	65+ years	189 (37.01)	*0.04*	*0.03*
		2.0	*(0.03,0.05)*	*(0.02,0.05)*
			P < .001	*P < .001*
**Sex**				
	Women	1067 (79.05)	ref	ref
		1.1		
	Men	1018 (78.96)	0.99	1.16
		1.1	(0.83,1.19)	(0.93,1.46)
			P = .95	*P* = .19
**Social grade**				
	ABC1	1222 (86.26)	ref	ref
		0.9		

	C2DE	863 (70.59)	*0.38*	*0.31*
		1.3	*(0.32,0.46)*	*(0.25,0.40)*
			P < .001	*P < .001*
**Disability status**				
	No activity or work limiting disability	1852 (84.48)	ref	ref
		0.8		
	Activity or work limiting disability	233 (52.18)	*0.20*	*0.45*
		2.2	*(0.16,0.25)*	*(0.35,0.57)*
			P < .001	*P < .001*
**Location**				
	Rural	270 (76.83)	ref	ref
		1.9		
	Urban	1815 (79.34)	1.16	0.96
		0.8	(0.92,1.46)	(0.73,1.28)
			P = .22	*P* = .79
**UK home nation**				
	England	1715 (79.54)	ref	ref
		0.9		
	Northern Ireland	58 (80.08)	1.03	1.05
		1.9	(0.80,1.33)	(0.76,1.44)
			P = .79	*P* = .78
	Scotland	179 (76.95)	0.86	0.93
		2.2	(0.66,1.12)	(0.68,1.28)
			P = .27	*P* = .65
	Wales	96 (73.06)	*0.70*	0.80
		2.3	*(0.54,0.90)*	(0.58,1.10)
			P = .006	*P* = .16
