# Supplementary figures and images for: Physical Activity Surveillance Through Smartphone Apps and Wearable Trackers: Examining the UK Potential for Nationally Representative Sampling
Source: JMIR Mhealth Uhealth. 2019 Jan 29;7(1):e11898. doi: 10.2196/11898 (PMC6371078; doi:10.2196/11898)

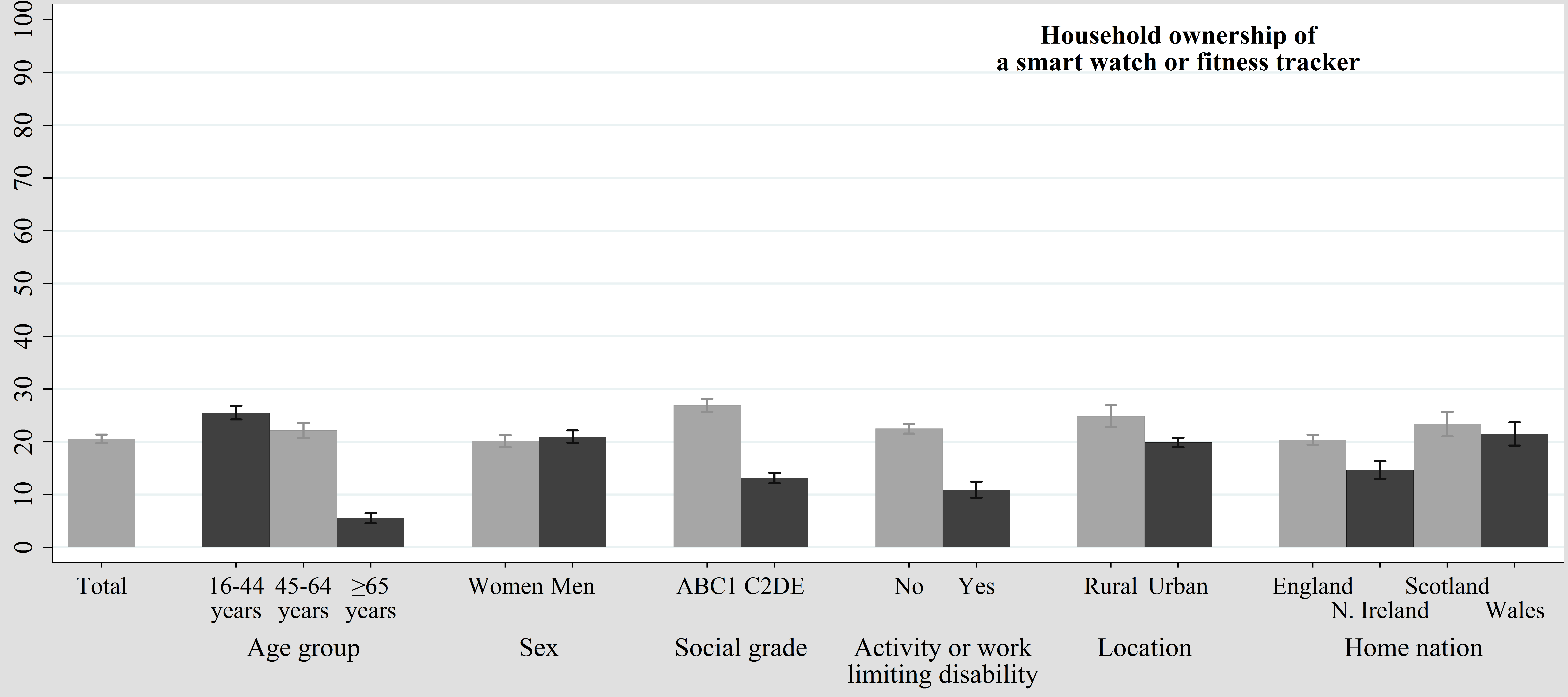

Supplement: Multimedia Appendix 3 [file mhealth_v7i1e11898_app3.png]

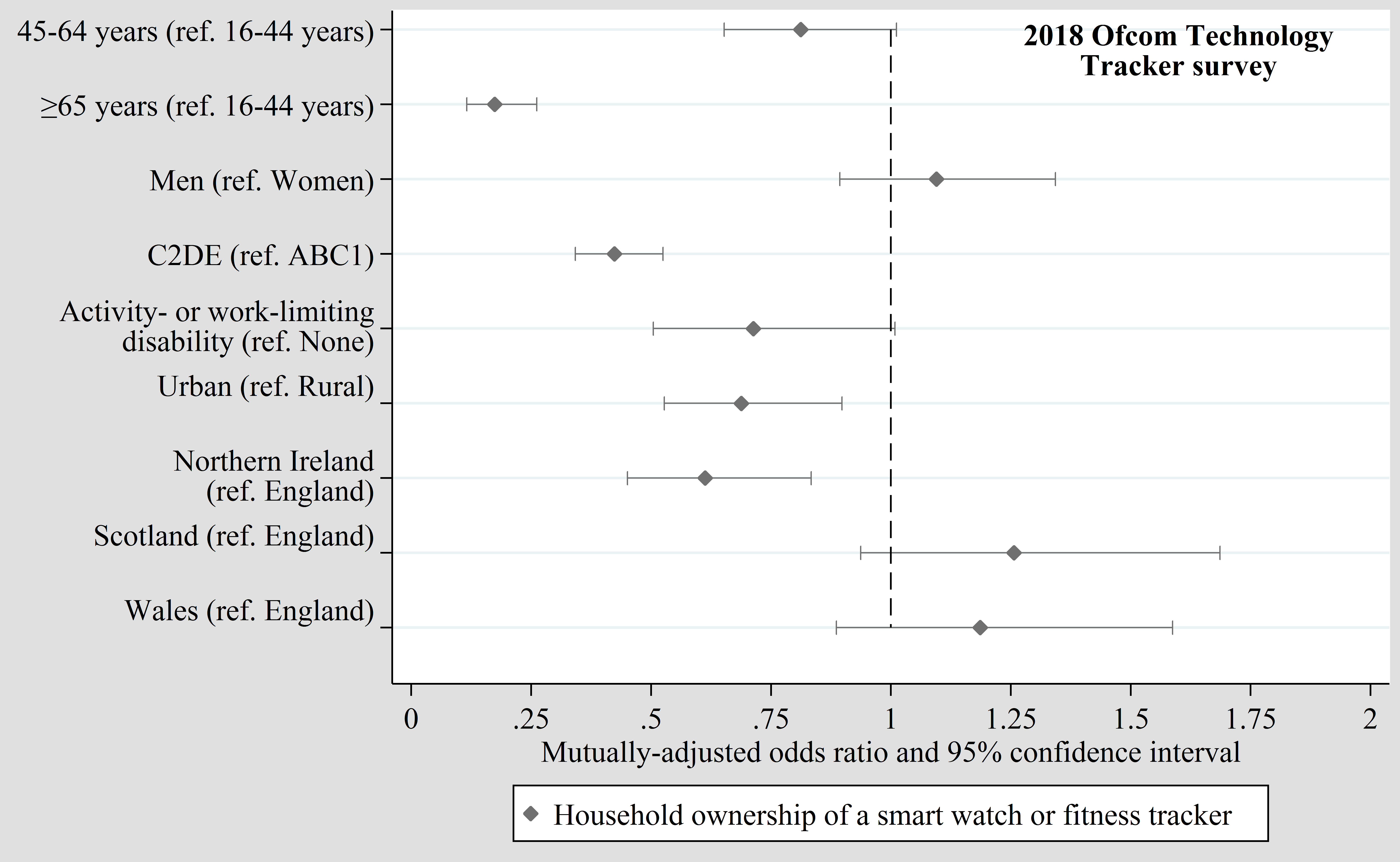

Supplement: Multimedia Appendix 5 [file mhealth_v7i1e11898_app5.png]

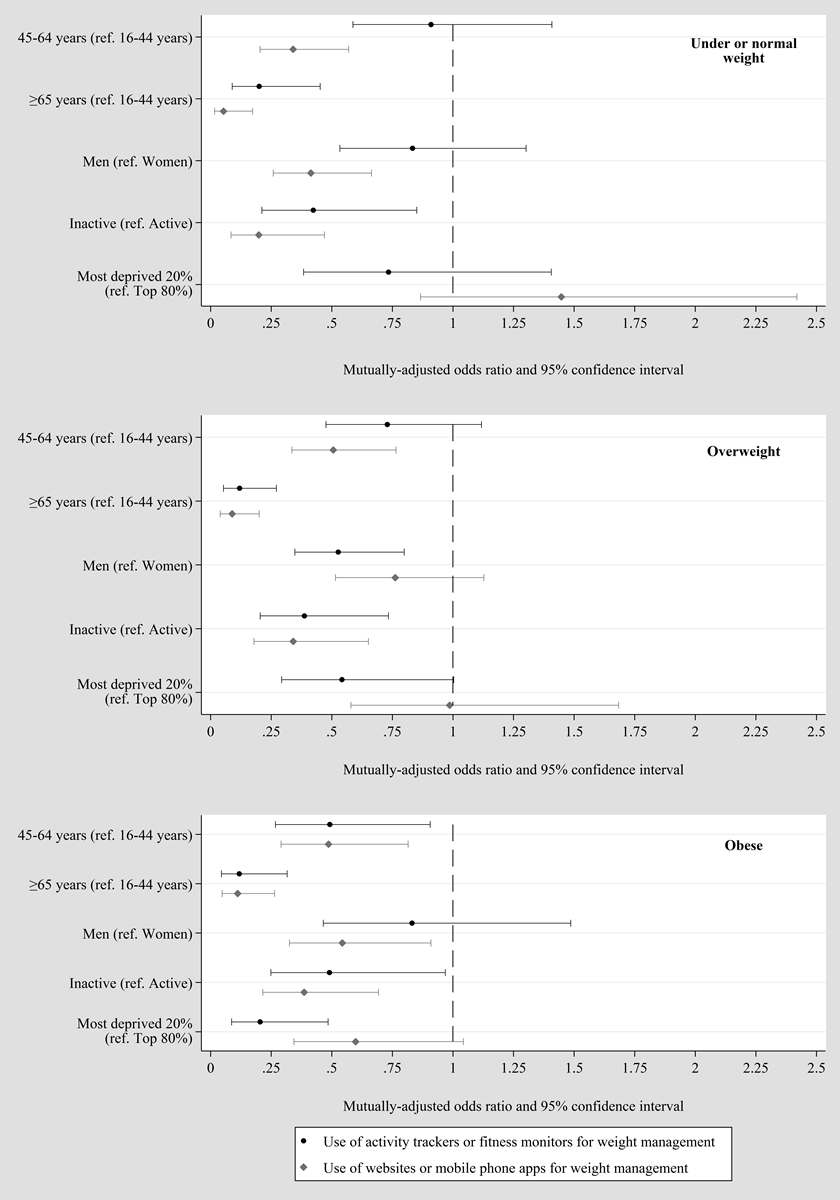

Supplement: Multimedia Appendix 7 [file mhealth_v7i1e11898_app7.png]
