# Supplementary material for: Physical Activity Surveillance Through Smartphone Apps and Wearable Trackers: Examining the UK Potential for Nationally Representative Sampling
Source: JMIR Mhealth Uhealth. 2019 Jan 29;7(1):e11898. doi: 10.2196/11898 (PMC6371078; doi:10.2196/11898)
Supplement: Multimedia Appendix 4 [file mhealth_v7i1e11898_app4.pdf]

Multimedia Appendix 4. Crude and mutually-adjusted odds ratios of reporting use of activity trackers or fitness monitors or websites or mobile phone applications for weight management, by socio-demographic characteristic in the 2016 Health Survey for England (unweighted N=4539, weighted N=4380).

		Weighted n (%) reporting outcome	Crude odds ratio	Mutually-adjusted odds ratio
		SE	95% confidence interval	95% confidence interval
			P-value	*P*-value
**Outcome: Use of activity trackers or fitness monitors for weight management**				
**Age group**				
	16-44 years	175 (8.69)	ref	ref
		0.7		
	45-64 years	98 (6.96)	0.79	*0.73*
		0.7	(0.60,1.03)	*(0.55,0.96)*
			P = .08	*P = .02*
	65+ years	13 (1.37)	*0.15*	*0.15*
		0.3	*(0.09,0.24)*	*(0.09,0.24)*
			P < .001	*P < .001*
**Sex**				
	Women	158 (7.20)	ref	ref
		0.5		
	Men	128 (5.85)	0.80	*0.71*
		0.6	(0.61,1.04)	*(0.54,0.93)*
			P = .10	*P = .01*
**Physical activity**				
	Active	255 (7.74)	ref	ref
		0.5		
	Inactive	31 (2.87)	*0.35*	*0.43*
		0.5	*(0.24,0.51)*	*(0.29,0.63)*
			P < .001	*P < .001*
**Deprivation**				
	Top 80%	253 (7.15)	ref	ref
		0.5		
	Most deprived 20%	33 (3.93)	*0.53*	*0.49*
		0.7	*(0.36,0.78)*	*(0.32,0.73)*
			P = .001	*P = .001*
**Body mass index**				
	Under/normal weight	116 (5.74)	0.77	*0.56*
		0.6	(0.54,1.09)	*(0.38,0.81)*
			P = .14	*P = .002*

Overweight	107 (7.13)	0.97	0.88
	0.7	(0.69,1.37)	(0.61,1.26)
		P = .87	*P* = .48
Obese	63 (7.33)	ref	ref
	1.0		
**Outcome: Use of websites or mobile phone applications for weight management**			
**Age group**			
16-44 years	275 (13.66)	ref	ref
	0.9		
45-64 years	101 (7.16)	*0.49*	*0.45*
	0.7	*(0.38,0.63)*	*(0.35,0.59)*
		P < .001	*P < .001*
65+ years	12 (1.25)	*0.08*	*0.08*
	0.3	*(0.05,0.13)*	*(0.05,0.14)*
		P < .001	*P < .001*
**Sex**			
Women	230 (10.49)	ref	ref
	0.7		
Men	157 (7.22)	*0.66*	*0.57*
	0.7	*(0.52,0.85)*	*(0.44,0.73)*
		P = .001	*P < .001*
**Physical activity**			
Active	353 (10.73)	ref	ref
	0.6		
Inactive	35 (3.20)	*0.27*	*0.31*
	0.5	*(0.19,0.40)*	*(0.21,0.45)*
		P < .001	*P < .001*
**Deprivation**			
Top 80%	308 (8.69)	ref	ref
	0.5		
Most deprived 20%	80 (9.55)	1.11	0.99
	1.2	(0.83,1.49)	(0.72,1.36)
		P = .49	*P* = .93
**Body mass index**			
Under/normal weight	154 (7.63)	*0.67*	*0.45*
	0.7	*(0.50,0.91)*	*(0.33,0.62)*
		P = .010	*P < .001*
Overweight	140 (9.31)	0.84	0.78
	0.8	(0.62,1.13)	(0.56,1.07)

			P = .25	*P* = .12
	Obese	94 (10.93)	ref	ref
		1.1		
