# Supplementary material for: Physical Activity Surveillance Through Smartphone Apps and Wearable Trackers: Examining the UK Potential for Nationally Representative Sampling
Source: JMIR Mhealth Uhealth. 2019 Jan 29;7(1):e11898. doi: 10.2196/11898 (PMC6371078; doi:10.2196/11898)
Supplement: Multimedia Appendix 6 [file mhealth_v7i1e11898_app6.pdf]

Multimedia Appendix 6 Crude and mutually-adjusted odds ratios of reporting use of activity trackers or fitness monitors or websites or mobile phone applications for weight management, by socio-demographic characteristic, stratified by body mass index, in the 2016 Health Survey for England (N=4539).

| | | Weighted n (%) reporting outcome | Crude odds ratio | Mutually-adjusted odds ratio |
|---|---|---|---|---|
| | | SE | 95% confidence interval | 95% confidence interval |
| | | | *P*-value | *P*-value |
| **Under/normal weight** | | | | |
| **Outcome: Use of activity tracker for weight management** | | | | |
| **Age group** | | | | |
| | 16-44 years | 78 (7.00) | ref | ref |
| | | 0.9 | | |
| | | | | |
| | 45-64 years | 32 (6.32) | 0.90 | 0.91 |
| | | 1.0 | (0.58,1.39) | (0.59,1.41) |
| | | | *P* = .63 | *P* = .67 |
| | 65+ years | 5 (1.29) | *0.17* | *0.20* |
| | | 0.5 | *(0.08,0.39)* | *(0.09,0.45)* |
| | | | *P < .001* | *P < .001* |
| **Sex** | | | | |
| | Women | 68 (6.14) | ref | ref |
| | | 0.7 | | |
| | | | | |
| | Men | 47 (5.24) | 0.85 | 0.83 |
| | | 0.9 | (0.54,1.32) | (0.53,1.30) |
| | | | *P* = .46 | *P* = .42 |
| **Physical activity** | | | | |
| | Active | 106 (6.59) | ref | ref |
| | | 0.7 | | |
| | | | | |
| | Inactive | 9 (2.31) | *0.33* | *0.42* |
| | | 0.7 | *(0.17,0.66)* | *(0.21,0.85)* |
| | | | *P = .002* | *P = .02* |
| **Deprivation** | | | | |
| | Top 80% | 101 (5.98) | ref | ref |
| | | 0.6 | | |
| | | | | |
| | Most deprived 20% | 14 (4.45) | 0.73 | 0.73 |
| | | 1.3 | (0.38,1.39) | (0.38,1.41) |
| | | | *P* = .34 | *P* = .35 |

| | | | | |
|---|---|---|---|---|
| **Outcome: Use of websites or mobile phone applications for weight management** | | | | |
| **Age group** | | | | |
| | 16-44 years | 129 (11.58) | ref | ref |
| | | 1.2 | | |
| | | | | |
| | 45-64 years | 22 (4.34) | *0.35* | *0.34* |
| | | 0.9 | *(0.21,0.57)* | *(0.20,0.57)* |
| | | | *P < .001* | *P < .001* |
| | 65+ years | 2 (0.55) | *0.04* | *0.05* |
| | | 0.3 | *(0.01,0.13)* | *(0.02,0.17)* |
| | | | *P < .001* | *P < .001* |
| **Sex** | | | | |
| | Women | 110 (9.86) | ref | ref |
| | | 1.0 | | |
| | | | | |
| | Men | 44 (4.86) | *0.47* | *0.41* |
| | | 1.0 | *(0.30,0.74)* | *(0.26,0.66)* |
| | | | *P = .001* | *P < .001* |
| **Physical activity** | | | | |
| | Active | 147 (9.14) | ref | ref |
| | | 0.9 | | |
| | | | | |
| | Inactive | 6 (1.56) | *0.16* | *0.20* |
| | | 0.6 | *(0.07,0.37)* | *(0.08,0.47)* |
| | | | *P < .001* | *P < .001* |
| **Deprivation** | | | | |
| | Top 80% | 122 (7.18) | ref | ref |
| | | 0.7 | | |
| | | | | |
| | Most deprived 20% | 32 (10.04) | 1.44 | 1.45 |
| | | 2.0 | (0.89,2.35) | (0.87,2.42) |
| | | | *P* = .14 | *P* = .16 |
| **Overweight** | | | | |
| **Outcome: Use of activity tracker for weight management** | | | | |
| **Age group** | | | | |
| | 16-44 years | 60 (10.29) | ref | ref |
| | | 1.4 | | |
| | | | | |
| | 45-64 years | 43 (7.70) | 0.73 | 0.73 |
| | | 1.1 | (0.48,1.11) | (0.48,1.12) |
| | | | *P* = .14 | *P* = .15 |
| | 65+ years | 5 (1.29) | *0.11* | *0.12* |
| | | 0.5 | *(0.05,0.25)* | *(0.05,0.27)* |
| | | | *P < .001* | *P < .001* |
| **Sex** | | | | |

| | | | | |
|---|---|---|---|---|
| | Women | 57 (9.13) | ref | ref |
| | | 1.1 | | |
| | | | | |
| | Men | 51 (5.73) | *0.60* | *0.53* |
| | | 0.9 | *(0.40,0.91)* | *(0.35,0.80)* |
| | | | *P = .02* | *P = .003* |
| **Physical activity** | | | | |
| | Active | 97 (8.62) | ref | ref |
| | | 0.9 | | |
| | | | | |
| | Inactive | 10 (2.66) | *0.29* | *0.39* |
| | | 0.8 | *(0.16,0.54)* | *(0.20,0.73)* |
| | | | *P < .001* | *P = .004* |
| **Deprivation** | | | | |
| | Top 80% | 94 (7.77) | ref | ref |
| | | 0.8 | | |
| | | | | |
| | Most deprived 20% | 13 (4.52) | 0.56 | 0.54 |
| | | 1.2 | (0.31,1.02) | (0.29,1.00) |
| | | | *P* = .06 | *P* = .05 |
| | | | | |
| **Outcome: Use of websites or mobile phone applications for weight management** | | | | |
| **Age group** | | | | |
| | 16-44 years | 90 (15.42) | ref | ref |
| | | 1.8 | | |
| | | | | |
| | 45-64 years | 45 (8.18) | *0.49* | *0.51* |
| | | 1.2 | *(0.32,0.74)* | *(0.34,0.77)* |
| | | | *P = .001* | *P = .001* |
| | 65+ years | 5 (1.36) | *0.08* | *0.09* |
| | | 0.5 | *(0.03,0.17)* | *(0.04,0.20)* |
| | | | *P < .001* | *P < .001* |
| **Sex** | | | | |
| | Women | 62 (10.01) | ref | ref |
| | | 1.2 | | |
| | | | | |
| | Men | 78 (8.82) | 0.87 | 0.76 |
| | | 1.2 | (0.59,1.28) | (0.51,1.13) |
| | | | *P* = .48 | *P* = .17 |
| **Physical Activity** | | | | |
| | Active | 128 (11.33) | ref | ref |
| | | 1.1 | | |
| | | | | |
| | Inactive | 12 (3.23) | *0.26* | *0.34* |
| | | 0.9 | *(0.14,0.49)* | *(0.18,0.65)* |

| | | | | |
|---|---|---|---|---|
| | | | *P* < .001 | *P* = .001 |
| **Deprivation** | | | | |
| | Top 80% | 112 (9.27) | ref | ref |
| | | 0.9 | | |
| | | | | |
| | Most deprived 20% | 28 (9.49) | 1.03 | 0.99 |
| | | 2.0 | (0.62,1.70) | (0.58,1.68) |
| | | | *P* = .92 | *P* = .96 |
| **Obese** | | | | |
| **Outcome: Use of activity tracker for weight management** | | | | |
| **Age group** | | | | |
| | 16-44 years | 37 (11.71) | ref | ref |
| | | 2.1 | | |
| | | | | |
| | 45-64 years | 23 (6.70) | *0.54* | *0.49* |
| | | 1.4 | *(0.30,0.98)* | *(0.27,0.91)* |
| | | | *P = .04* | *P = .02* |
| | 65+ years | 3 (1.67) | *0.13* | *0.12* |
| | | 0.7 | *(0.05,0.34)* | *(0.04,0.32)* |
| | | | *P < .001* | *P < .001* |
| **Sex** | | | | |
| | Women | 33 (7.17) | ref | ref |
| | | 1.2 | | |
| | | | | |
| | Men | 30 (7.51) | 1.05 | 0.83 |
| | | 1.6 | (0.60,1.85) | (0.47,1.49) |
| | | | *P* = .86 | *P* = .53 |
| **Physical activity** | | | | |
| | Active | 51 (9.33) | ref | ref |
| | | 1.4 | | |
| | | | | |
| | Inactive | 12 (3.83) | *0.39* | *0.49* |
| | | 1.1 | *(0.20,0.76)* | *(0.25,0.97)* |
| | | | *P = .006* | *P = .04* |
| **Deprivation** | | | | |
| | Top 80% | 58 (9.09) | ref | ref |
| | | 1.3 | | |
| | | | | |
| | Most deprived 20% | 6 (2.42) | *0.25* | *0.20* |
| | | 0.9 | *(0.11,0.57)* | *(0.09,0.48)* |
| | | | *P = .001* | *P < .001* |
| | | | | |
| **Outcome: Use of websites or mobile phone applications for weight management** | | | | |
| **Age group** | | | | |
| | 16-44 years | 56 (17.81) | ref | ref |

| | | | | |
|---|---|---|---|---|
| | | 2.5 | | |
| | | | | |
| | 45-64 years | 33 (9.75) | *0.50* | *0.49* |
| | | 1.6 | *(0.30,0.81)* | *(0.29,0.81)* |
| | | | *P = .006* | *P = .006* |
| | 65+ years | 5 (2.34) | *0.11* | *0.11* |
| | | 0.9 | *(0.05,0.26)* | *(0.05,0.26)* |
| | | | *P < .001* | *P < .001* |
| **Sex** | | | | |
| | Women | 58 (12.63) | ref | ref |
| | | 1.6 | | |
| | | | | |
| | Men | 36 (8.95) | 0.68 | *0.54* |
| | | 1.7 | (0.42,1.11) | *(0.33,0.91)* |
| | | | *P* = .12 | *P = .02* |
| | | | | |
| | Active | 78 (14.16) | ref | ref |
| | | 1.6 | | |
| | | | | |
| | Inactive | 16 (5.26) | *0.34* | *0.39* |
| | | 1.3 | *(0.19,0.59)* | *(0.22,0.69)* |
| | | | *P < .001* | *P = .001* |
| **Deprivation** | | | | |
| | Top 80% | 74 (11.64) | ref | ref |
| | | 1.4 | | |
| | | | | |
| | Most deprived 20% | 20 (8.94) | 0.74 | 0.60 |
| | | 1.9 | (0.44,1.25) | (0.34,1.04) |
| | | | *P* = .27 | *P* = .07 |
